# Influence of pressurization rate and mode on cell damage of *Escherichia coli* and *Staphyloccocus aureus* by high hydrostatic pressure

**DOI:** 10.3389/fmicb.2023.1108194

**Published:** 2023-03-02

**Authors:** Dong Yang, Renjie Li, Peng Dong, Lei Rao, Yongtao Wang, Xiaojun Liao

**Affiliations:** ^1^College of Food Science and Nutritional Engineering, China Agricultural University, Beijing, China; ^2^National Engineering Research Center for Fruit and Vegetable Processing, Beijing, China; ^3^Key Lab of Fruit and Vegetable Processing, Ministry of Agriculture and Rural Affairs, Beijing, China; ^4^Beijing Key Laboratory for Food Non-Thermal Processing, Beijing, China

**Keywords:** high hydrostatic pressure, pressurization rate, pressurization mode, cell damage, *Escherichia coli*, *Staphyloccocus aureus*

## Abstract

As a non-thermal technology, high hydrostatic pressure (HHP) has been widely investigated for inactivating microorganisms in food. Few studies have been presented on the pressurization/depressurization rate and mode of microbial inactivation. In this study, effect of pressurization rate and mode on *Escherichia coli* and *Staphylococcus aureus* cell damage during HHP treatment was investigated. The results showed that fast pressurization + linear mode (FL) treatment has the best bactericidal effect on *E. coli* and *S. aureus*, followed by fast pressurization + stepwise mode (FS) and slow pressurization + stepwise mode (SS) treatments. FL treatment caused more morphological damage to the cell wall, cell membrane, and cytoplasmic components compared with FS and SS treatment detected by SEM and TEM. Additionally, the damage to membrane permeability of them was also enhanced after FL treatment. Therefore, our results indicated that FL treatment could be applied to enhance the bactericidal effect of HHP on bacteria by increasing the damage to cell morphological structure and membrane integrity.

## 1. Introduction

Thermal sterilization is the typical traditional food sterilization process and is widely used (Soni et al., 2020). It can effectively kill bacteria in various foods such as rice (Auksornsri et al., 2018), eggs (Soni et al., 2020), and meat products (Romaniw et al., 2022). However, high temperature introduced by thermal sterilization may affect the characteristics of food, such as flavor, color, or nutritional value (Wang et al., 2018; Zhang et al., 2022). As a novel non-thermal technology, high hydrostatic pressure (HHP) has been widely investigated for inactivating the microorganisms in food (Yang et al., 2021). In this technology, food is subjected to high pressure intensities of 100 to 1,000 MPa utilizing a liquid pressure transfer medium (such as H_2_O) in a flexible container at room or lower temperatures to accomplish microbial inactivation (Sehrawat et al., 2021). Unlike thermal processing, HHP could preserve most low molecular weight substances contributing to quality attributes, such as color, flavor, biological activity and nutritional value (Oey et al., 2008; Wang et al., 2016). HHP was a safe, energy-efficient and waste-free technology, and can be used for both liquid and solid products (Priyadarshini et al., 2019).

The microorganism inactivation efficacy of HHP is affected by a number of factors, such as the magnitude of pressure, temperature, pressure holding time, pH, water activity and species of microorganism (Huppertz et al., 2006; Agregán et al., 2021). Few studies have been presented on the pressurization/depressurization rate and mode of microbial inactivation. However, the available studies on this topic were quite contradictory and there is room for more research on this aspect (Syed et al., 2016). Herdegen (1998) reported that compared with a pressurization rate of 50 MPa/s and pressure relief rate of 400 MPa/min, a faster pressurization rate of 400 MPa/min had a better bactericidal effect. They also proposed that a slow ramp during compression might induce a stress response of microbial cells and hence lead to a lower inactivation effect by HHP. In the same way, studies have shown that a 600 MPa/min pressurization rate and 15,000 MPa/min pressure relief rate had a better killing effect than a slow increase and discharge rate (60 MPa/min, 300 MPa/min; Chapleau et al., 2006). In addition, Meloni (2019) reported that a pressurization rate of 400 MPa/min and relief rate of 50 MPa more effectively inhibited the growth of *Listeria monocytogenes* than a pressurization rate of 50 MPa/min and relief rate of 400 MPa/min. These studies showed that the faster pressure-increasing process of HHP treatment might improve the bactericidal effect on microorganisms, giving new insight into improving the HHP bactericidal effect.

Our previous studies investigated the bactericidal effect of HHP on different pressures, pressurization rates and pressurization modes on purple sweet potato juice (Wang et al., 2012, 2013), and found that linear pressurization of 120 MPa/min has a better sterilizing effect compared with a rate of 120 MPa/min stepwise model and a rate of 60 MPa/min stepwise model pressurization during high pressure treatment. This result indicated that a faster pressurization rate resulted in a greater reduction of microorganisms compared with a slower pressurization rate under the same pressurization conditions (Wang et al., 2013). Moreover, different stress response pathways of *Escherichia coli* O157:H7 under different pressurization modes and rates were analyzed by studying GroEL-interacting proteins. We proposed a possible mechanism that GroEL interacts with different proteins under different pressurization modes and rates to protect cells from high-pressure stress (Dong et al., 2021). However, the reasons for the different bactericidal effects caused by different pressurization rates and modes are still unclear.

*Escherichia coli* O157:H7 and *Staphyloccocus aureus* are two important bacterial pathogens frequently implicated in foodborne outbreaks, and representatives of Gram-negative and Gram-positive microbes, respectively (Llamazares et al., 2019). Human infection with *E. coli* O157:H7 can cause bloody diarrhea and kidney failure (Gally and Stevens, 2017). *S. aureus* infection can affect the skin, bloodstream, bone tissue, or eyes and lead to life-threatening diseases such as endocarditis, pneumonia, toxic shock syndrome or keratitis (Anwar et al., 2009). Both can be spread in a diversity of ways, but foodborne spread is the most important route and occurs primarily through the consumption of contaminated drinking water and raw foods (Karch et al., 2005). Hence, research on the bactericidal mechanism of different pressurization rates and modes during HHP treatment for *E. coli* O157:H7 and *S. aureus* is of great importance for food processing and storage.

In this research, the damage to microbial cell morphology, structure and membrane permeability caused by different pressurization rates and modes was investigated, and the reason for the FL treatment causing greater damage to the microbial cell was proposed. This study provides a theoretical basis for the mechanism of high-pressure sterilization and a new insight for designing HHP parameters to control microorganisms in food.

## 2. Materials and methods

### 2.1. Microbial preparation

*Escherichia coli* O157:H7 NCTC 12900, a well characterized *stx*-negative strain, was obtained from the National Culture Type Collection (Colindale, London, United Kingdom). *S. aureus* (CGMCC 1.1861) was obtained from China General Microbiological Culture Collection Center (CGMCC, Beijing, China). The strains of *E. coli* and *S. aureus* were stocked in tryptic soy broth (TSB) and nutrient broth (NB) with 25% glycerol at −80°C. Overnight cultures of *E. coli* and *S. aureus* were prepared by selecting an activated single colony of strains and growing it in 5 mL of TSB and NB broth in a shaking incubator at 37°C. These overnight cultures were transferred to fresh medium at a dilution of 1:100 and grown to the exponential phase (OD_600_ = 0.63, 0.71) for further experiments. Exponential-phase cells of *E. coli* or *S. aureus* were centrifuged at 8340 *g* for 10 min at 4°C, and the pellets were washed twice with sterile normal saline and then resuspended in normal saline. The cell concentration of *E. coli* or *S. aureus* in the resulting suspensions was approximately 1 × 10^8^ CFU/mL.

### 2.2. High hydrostatic pressure treatment

**Figure 1:**
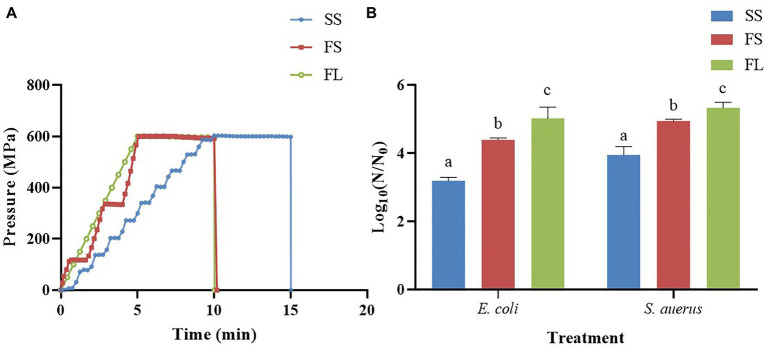
Effects of pressurization rates and modes on inactivation of *E. coli* (250 MPa/5 min) and *S. aureus* (450 MPa/5 min). **(A)** Pressure profiles of SS, FS and FL as a function of time. SS was pressurized at a rate of 60 MPa/min by a one-way intensifier with a come-up-time of 10 min. FS was pressurized at 120 MPa/min by a one-way intensifier with a come-up-time of 5 min. FL was pressurized at 120 MPa/min by a two-way intensifier with a come-up-time of 5 min. The depressurization rate was fixed in SS, FS and FL (<3 s). **(B)** Effects of pressurization rates and modes on inactivation. Different letters in the same strain shows significant difference.

HHP treatment with the procedures described previously (Wang et al., 2013), there were three different hydrostatic pressurization units (HHP-600, HHP-650 and HHP-700, Baotou Kefa Co., Ltd., Inner Mongolia, China) and different external high pressure intensifiers were used to achieve different pressurization rate and mode during HHP treatment. The parameters of HHP were shown in Figure 1A; Table 1. The depressurization rate was the same for all treatments and H_2_O was used as the pressure-transmitting fluid. The samples were treated by HHP at 250 MPa/5 min and 450 MPa/5 min for *E. coli* and *S. aureus*, respectively.

**Table 1:** Description of parameters of HHP.

Code	HHP unit	Number of intensifier	Type of intensifier	Average pressurization rate (MPa/min)	Pressurization mode	Depressurization
SS	HHP-600	1	One-way	60	Stepwise	<3 s
FS	HHP-650	1	One-way	120	Stepwise	<3 s
FL	HHP-700	1	Two-way	120	Linear	<3 s

The following codes were used for sample identification.

(1) Slow pressurization (60 MPa/min) + stepwise mode (one-way intensifier) = SS.

(2) Fast pressurization (120 MPa/min) + stepwise mode (one-way intensifier) = FS.

(3) Fast pressurization (120 MPa/min) + linear mode (two-way intensifier) = FL.

(4) Untreated = Control.

### 2.3. Viable cell count detection

The viable cell count after different treatments was detected by plate count method. Each sample was serially diluted with sterile normal saline, and each dilution was plated into duplicate plates. Plates were used for counting the viable *E. coli* and *S. aureus* cells after incubation at 37°C for 24 h. Log *N/N_0_* was calculated to determine the inactivation effect of HHP treatment, where *N_0_* was the number of initially viable cells in the untreated sample, and *N* was the corresponding number of viable cells after HHP treatment. The initial counts of the *E. coli* and *S. aureus* cells were 2.44 × 10^8^ CFU/mL and 3.01 × 10^8^ CFU/mL, respectively.

### 2.4. Scanning electron microscope and transmission electron microscope analysis

The SEM and TEM were used to observe the morphology and interior structure of exponential *E. coli* and *S. aureus* cells untreated or treated by HHP with the procedures described previously (Zhao et al., 2016).

### 2.5. CLSM image analysis

The untreated and HHP-treated *E. coli* and *S. aureus* cells were centrifuged at 8340 *g* for 10 min. The supernatant was removed and the pellet was washed twice with normal saline. Then, the cells were stained by a Live/Dead BacLight bacterial viability kit according to the manufacturer’s instructions (Molecular Probes Inc., Eugene, OR, United States). The Propidium Iodide (Cat. No. P8080) was purchased from Beijing Solarbio Science & Technology Co., Ltd. Bacteria solution was incubated with dye mixture (SYTO 9:PI = 1:1) at room temperature in darkness for 15 min, with a final concentration of 5 μM SYTO 9 and 30 μM PI. The cell suspension after staining was placed between a slide and an 18 mm square coverslip. Samples were examined under a Zeiss LSM710 CLSM (Carl Zeiss MicroImaging GmbH, Jena, Germany). In all cases, a 63× objective was used with immersion oil.

### 2.6. FCM analysis

Samples were washed twice and diluted 100 times in PBS containing SYTO 9 and propidium iodide. Then, these samples were incubated for 15 min in the dark at room temperature and analyzed with a FACS Calibur flow cytometer (Becton Dickinson Immunocytometry Systems, New Jersey, United States). The green and red fluorescence of SYTO 9 and PI were collected in the FL1 and FL2 channels with 502 nm and 613 nm long-pass filters, respectively. Log phase bacteria were used to focus population gates around *E. coli* or *S. aureus* cells that were alive and had intact cell membrane. Data were collected using CellQuest software (Becton Dickinson, San Jose, CA, United States; Pan et al., 2019).

### 2.7. Statistical analysis

Each experiment was carried out at least in triplicate. Graph drawing and analysis of variance (ANOVA) were conducted using Graphpad prism 8 software. The data were expressed as mean value ± S.D. *p* < 0.05 was considered statistically significant for all assays.

## 3. Results and discussion

### 3.1. Effect of pressurization rate and mode on inactivation of *Escherichia coli* and *Staphyloccocus aureus*

To find the effect of pressurization rate and mode on the inactivation of microorganisms, Gram-negative *E. coli* and Gram-positive *S. aureus* were treated with SS, FS and FL HHP, respectively. The results were shown in Figure 1B. SS, FS, and FL treatments caused 3.19, 4.39 and 5.02 logs reduction in *E.coli* respectively, which showed that FL treatment had the best bactericidal effect, followed by FS and SS treatments. This phenomenon was also found in *S. aureus*. These results proved that the pressurization process could significantly affect the inactivation efficiency of HHP on the microorganism, and the FL exhibited the highest bactericidal effect among these treatments. It was consistent with our previous study (Wang et al., 2013), which reported that FL treatment significantly enhanced pressure inactivation of total aerobic bacteria in purple sweet potato nectar. However, the log reduction of *E. coli* and *S. aureus* caused by the different HHP treatments (SS, FS, and FL) was not as significant as the log reduction of native microorganisms in purple sweet potato nectar (Wang et al., 2013). This may be due to the presence of microorganisms that were sensitive to different pressurization rates in the purple sweet potato nectar. Moreover, the pH value of purple sweet potato nectar (pH 3.7) was significantly lower than physical saline. Since the pressure-induced inactivation of vegetative cells is accelerated at low pH values, compared to neutral pH value (Aganovic et al., 2021).

### 3.2. Cellular morphology and interior structure of *Escherichia coli* and *Staphyloccocus aureus* treated by different pressurization rates and modes

**Figure 2:**
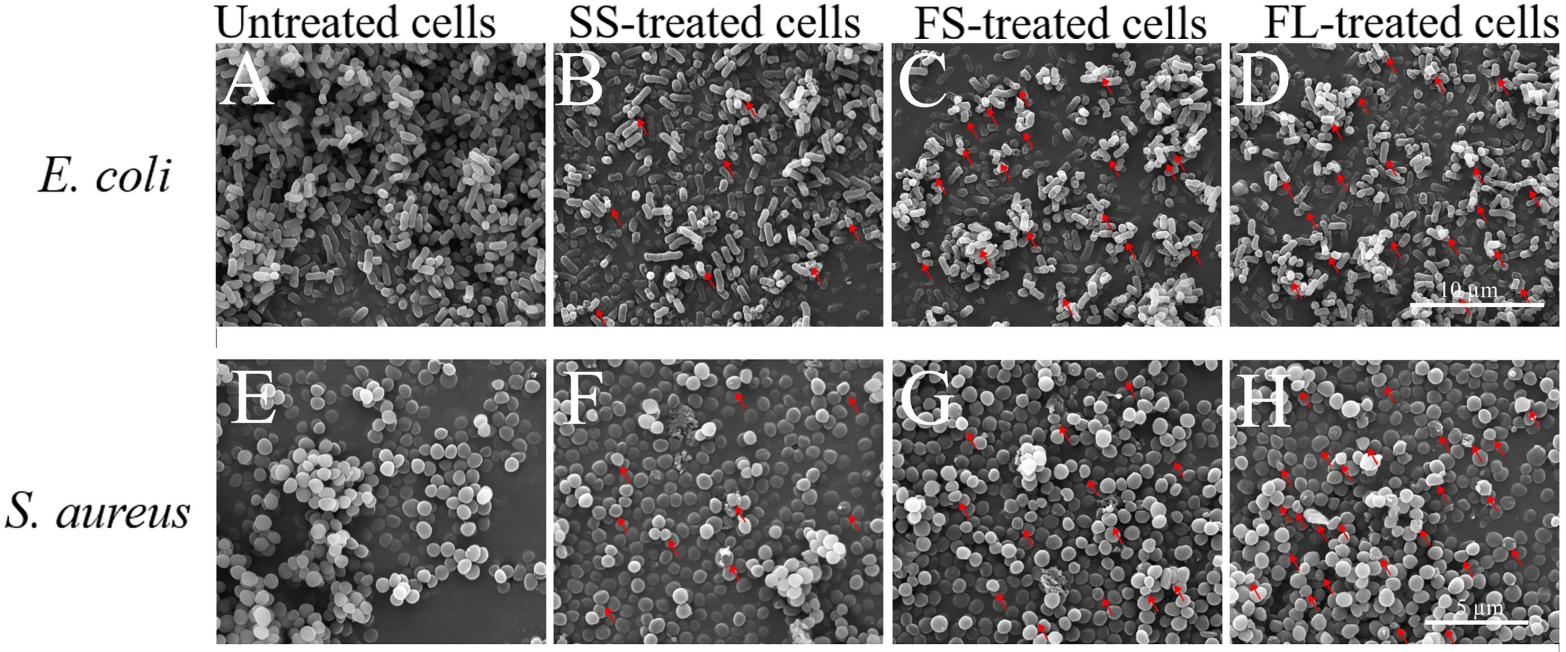
Scanning electron micrograph images of *E. coli* (10,000×, **A–D**) and *S. aureus* (15,000×, **E–H**) treated by high hydrostatic pressure (250 MPa/5 min and 450 MPa/5 min, respectively). **(A,E)** Control. **(B,F)** SS treatment. **(C,G)** FS treatment. **(D,H)** FL treatment. The arrows referred to the damage on the cell surface.

To visualize the effect of different HHP pressurization rates and modes on cellular morphology, the cells after SS, FS, and FL treatment were detected by SEM and TEM. As shown in Figure 2, the untreated *E. coli* cells were intact, showing regularly rod-shaped morphology with smooth surfaces (Figure 2A). After SS treatment, the surface of *E. coli* cells became rough and pitted (Figure 2B). There were many studies have reported the same phenotypes of *E. coli*, *L. monocytogenes*, *L. mesenteroides*, *S. typhimurium*, and *L. viridescens* after HHP treatment (Tholozan et al., 2000; Ritz et al., 2001; Kaletunc et al., 2004; Pilavtepe-Celik et al., 2008; Zhao et al., 2017). Moreover, the surface of a large fraction of FS-treated *E. coli* cells was deformed and wrinkled (Figure 2C). Notably, the cells after FL treatment were sunken or even ruptured (Figure 2D). FL treatment caused more severe damage to *E. coli* cells than SS and FS treatment, which supported the enhanced effect of FL treatment on *E. coli* inactivation.

The untreated *S. aureus* cells were intact, showing regularly sphere-shaped morphology with smooth surfaces (Figure 2E). After HHP treatment, only a small fraction of HHP-treated *S. aureus* cells (Figures 2F–H) were significantly deformed, which was not consistent with the change in *E. coli*. The different phenotypes of these two strains after HHP treatment may be attributed to the different cell structures between *E. coli* (Gram-negative bacteria) and *S. aureus* (Gram-positive bacteria). *S. aureus* has a thicker cell wall, which caused lower susceptibility to HHP (Elke et al., 2002; Wang et al., 2010; Li et al., 2016; Zhao et al., 2017). Moreover, the same as *E. coli*, FL-treated *S. aureus* cells (Figure 2H) exhibited more damage on their surface compared with SS-treated cells and FS-treated cells (Figures 2F,G).

**Figure 3:**
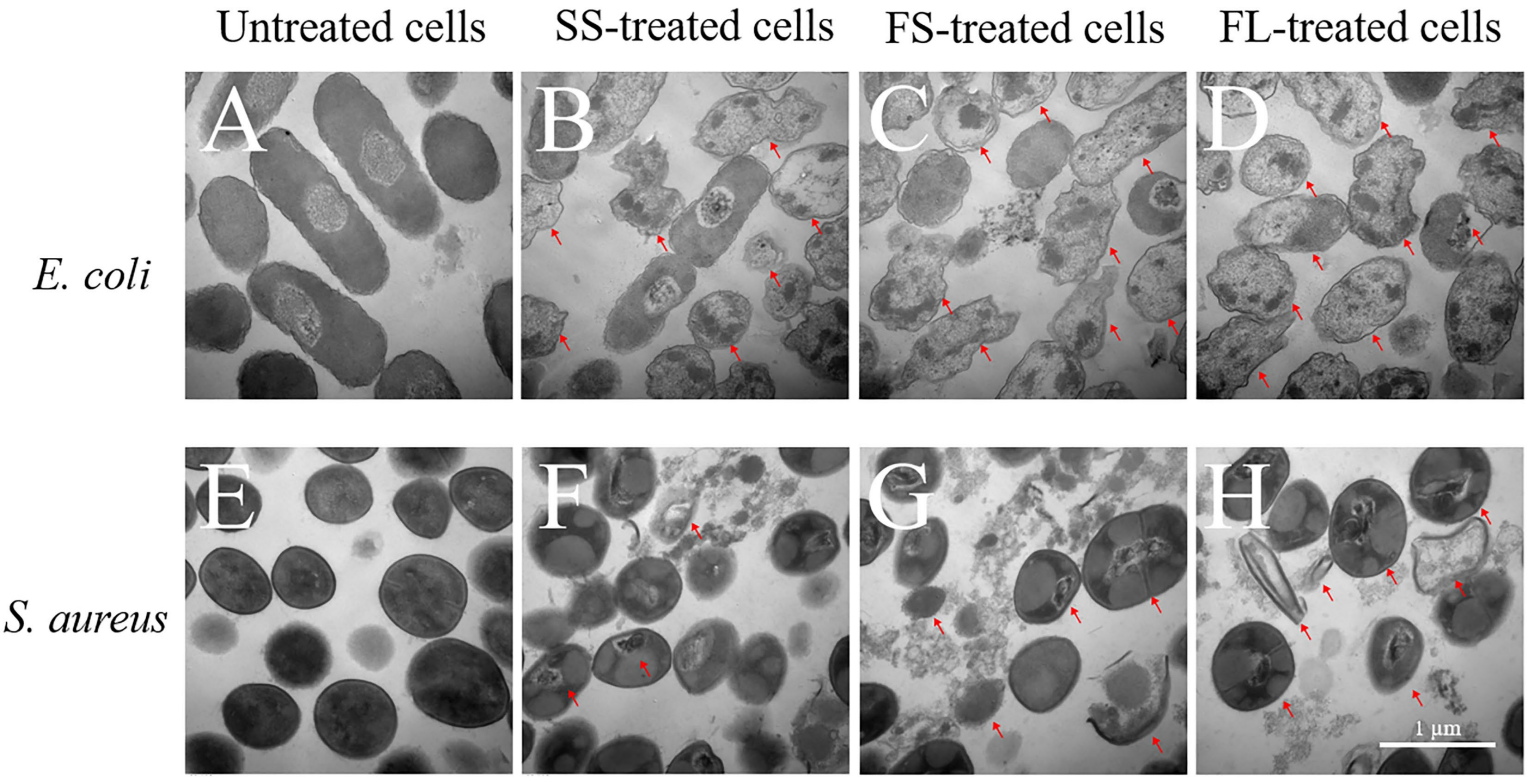
Transmission electron micrograph images of *E. coli* (40,000×, **A–D**) and *S. aureus* (50,000×, **E–H**) treated by high hydrostatic pressure (250 MPa/5 min and 450 MPa/5 min, respectively). **(A,E)** Control. **(B,F)** SS treatment. **(C,G)** FS treatment. **(D,H)** FL treatment. The arrows referred to the damaged cells.

Though FL treatment damaged the cell morphology more than SS and FS, it could not fully explain why FL treatment had the best bactericidal effect. Therefore, the interior structure of *E. coli* and *S. aureus* by SS, FS and FL treatment was investigated by TEM to analyze the possible enhancement mechanism of FL treatment. As shown in Figure 3A, the untreated *E. coli* showed clearly visible intracellular components, and the protoplasm was uniformly distributed. Oval dark regions of high electron density within the cell were observed, indicating a compact internal structure of the cell (Li et al., 2016). After SS treatment, only a small fraction of cells remained intact, and most of the cells were damaged by HHP. The distribution of protoplasm in the cell was no longer uniform, and some cells exhibited protoplasm aggregation or even cavities in the protoplasm (Figure 3B). After FS and FL treatment, few structurally intact cells were observed. The cell membranes bending deformation were more severe than SS treatment, and a large fraction of cells have the phenomenon of outflow of cellular contents or even ruptured (Figures 3C,D).

The untreated *S. aureus* cells were smooth spherical with a uniform distribution of protoplasm (Figure 3E). After SS treatment, most *S. aureus* cells still possess integrity of cellular shape and structure. A small fraction of cells showed the distribution of protoplasm inside the cell with intact membrane was no longer uniform or even aggregated (Figure 3F). After FS treatment, a portion of the cells was lysed and cytoplasm flowed out of the cells (Figure 3G). After FL treatment, the damage to the cell structure was more intensified compared with SS and FS treatment. In addition to the aggregation of protoplasts and the rupture of the cell membrane, part of the cell cytoplasm has been completely lost, leaving only the severely ruptured and deformed cell shell (Figure 3H).

SS, FS, and FL treatment could effectively destroy cell structure regardless of Gram-positive or Gram-negative bacteria. The disruption efficiency of FL treatment on cell structure was significantly higher than that of SS and FS treatment. This was consistent with the bactericidal effect of SS, FS, and FL treatments (Figure 1B). These results implied structure destruction caused by different treatments resulting in the difference in bactericidal effect. *S. aureus* revealed relatively better structural integrity after HHP treatment, though it was treated with a higher pressure than *E. coli*. This might be because Gram-positive bacteria have a thicker peptidoglycan layer and higher mechanical strength compared with Gram-negative bacteria (Syed et al., 2016), which is consistent with the results obtained in the TEM images (Figures 3A,E).

### 3.3. Membrane permeability of *Staphyloccocus aureus* and *Escherichia coli* cells treated by different pressurization rates and modes

The bactericidal effect of HHP on microorganisms is mainly by destroying the physiological function of cells, interrupting their multiplication and repair function (Wang et al., 2016). Cellular targets of high pressure treatment include outer membrane barrier properties, cytoplasmic membrane integrity, and membrane-bound enzyme activity. Pressure-induced rupture of the cell membrane and denaturation of membrane-bound enzymes may lead to the generation of reactive oxygen species and subsequent cell death caused by oxidative stress (Gänzle and Liu, 2015). Therefore, the cell membrane permeability was detected after different pressurization processes.

**Figure 4:**
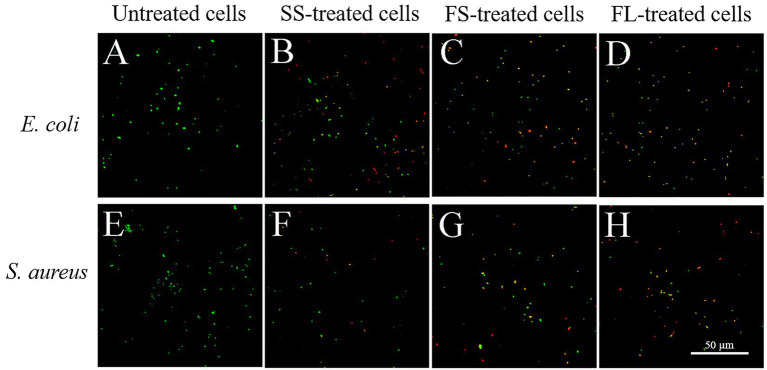
Confocal laser scanning microscope images of *E. coli* and *S. aureus* treated by high hydrostatic pressure (250 MPa/5 min and 450 MPa/5 min, respectively) and stained with SYTO 9 and PI. **(A,E)** Control. **(B,F)** SS treatment. **(C,G)** FS treatment. **(D,H)** FL treatment.

The cells were stained with SYTO 9 and PI to reveal the cell membrane permeability after different treatments. The results were shown in Figure 4, the untreated *E. coli* only could be staining with SYTO 9, indicating that their cell membrane was intact and possessed selective permeability (Figure 4A). However, a part of the cells was stained with PI after SS treatment (Figure 4B), and the proportion of cells that could be stained with PI increased after FS treatment (Figure 4C). After FL treatment, most of the cells were stained with PI and have red or yellow fluorescence (Figure 4D). The changes in membrane permeability of *S. aureus* after different pressurization processes were similar to those of *E. coli* (Figures 4E–H). These results indicated that different pressurization rates and modes had distinct changes in cell membrane permeability. FL treatment showed the most severe damage to the selective permeability of the membrane, which was consistent with SEM and TEM results.

**Figure 5:**
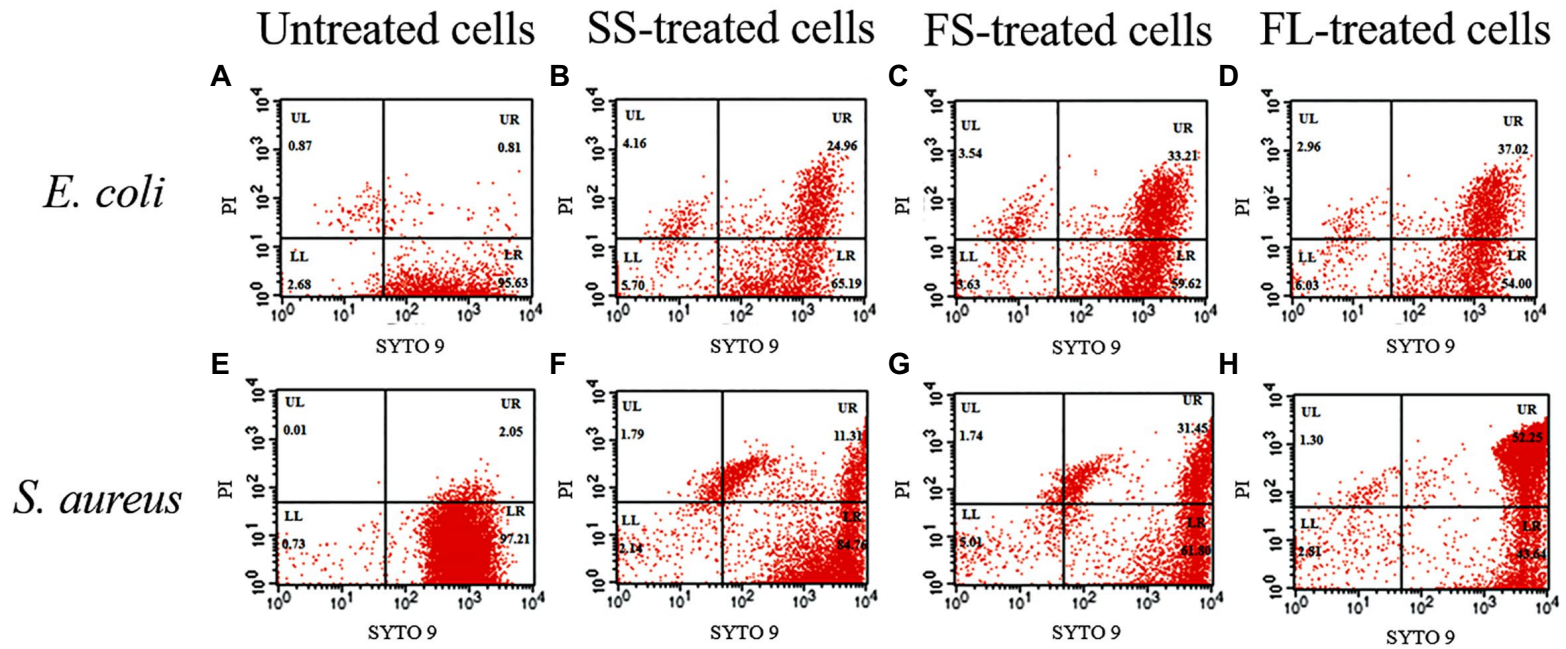
Flow cytometry analysis of *E. coli* and *S. aureus* treated by high hydrostatic pressure (250 MPa/5 min and 450 MPa/5 min, respectively) and stained with SYTO 9 and PI. **(A,E)** Control. **(B,F)** SS treatment. **(C,G)** FS treatment. **(D,H)** FL treatment.

In order to quantify the damage of the different treatments on cell membrane permeability, flow cytometry was used to detect the effect of different pressurization processes on the cell membrane integrity of *E. coli* and *S. aureus*. As shown in Figure 5, FL1 channel represents the green fluorescence channel of SYTO 9, and FL2 channel represents the red fluorescence channel of PI. The cells in the LR quadrant have an intact membrane with selective penetration, and the cell membrane in this UR quadrant is damaged and selective permeability is lost. The ratio of untreated *E. coli* cells in LR quadrant was 95.63%, indicating that most untreated cells have intact cell membranes with selective permeability (Figure 5A). However, this ratio was reduced to 65.19, 59.62 and 54.00% after SS, FS and FL treatment (Figures 5B–D), respectively. The ratios in LR quadrant of *S. aureus* after different pressurization processes were similar to those of *S. aureus*. The ratios in LR quadrant were reduced to 84.76, 61.80 and 43.64% after SS, FS and FL treatment (Figures 5F–H). The cell membrane integrity after SS, FS and FL treatments were consistent with the bactericidal effect of these treatments (Figures 1B, 5). Previous studies reported that destroying the membrane fluidity, integrity and other physiological functions of the cell membrane is a major mechanism in the HHP-mediated bactericidal effect (Considine et al., 2008). Our results further proved that fast rate and linear pressurization mode could promote the bactericidal effect of HHP by increasing the damage to membrane integrity.

## 4. Conclusion

In this study, the effect of three different HHP pressurization rates and modes on inactivation for both Gram-negative *E. coli* and Gram-positive *S. aureus* was investigated. We found that the pressurization process could significantly affect the inactivation efficiency of HHP on microorganisms and FL exhibited the strongest bactericidal effect followed by FS and SS. Cellular morphology analysis by SEM and TEM revealed that the disruption efficiency of FL treatment on cell structure is significantly higher than that of SS and FS. Moreover, the change of membrane permeability assessed by CLSM and flow cytometer showed that SS has the least damage to the membrane integrity, and FL had the most damage to the membrane integrity. Therefore, our results indicated that FL could promote the bactericidal effect of HHP by increasing the damage to cell morphological structure and membrane integrity. This improvement in the bactericidal effect of HHP treatment could lead to a reduction in the pressure holding time and pressure level required for microbial inactivation, and a reduction in the production cost of HHP-processed foods. Therefore, it is expected that the FL can be used in HHP-processed food with improved quality at a reasonable cost.

## Data availability statement

The original contributions presented in the study are included in the article/supplementary material, further inquiries can be directed to the corresponding authors.

## Author contributions

XL, YW, and LR planned and designed the research. YW, DY, RL, and PD performed experiments, data analysis, and wrote the manuscript. XL and YW contributed to manuscript revision, read and approved the submitted version. All authors contributed to the article and approved the submitted version.

## Funding

This study was financially supported by Project No. 2022YFD2100801 of the National Key Research and Development Program of China and the 2115 Talent Development Program of China Agricultural University.

## Conflict of interest

The authors declare that the research was conducted in the absence of any commercial or financial relationships that could be construed as a potential conflict of interest.

## Publisher’s note

All claims expressed in this article are solely those of the authors and do not necessarily represent those of their affiliated organizations, or those of the publisher, the editors and the reviewers. Any product that may be evaluated in this article, or claim that may be made by its manufacturer, is not guaranteed or endorsed by the publisher.
